# Supplementary material for: Acetyl-CoA flux regulates the proteome and acetyl-proteome to maintain intracellular metabolic crosstalk
Source: Nat Commun. 2019 Sep 2;10:3929. doi: 10.1038/s41467-019-11945-9 (PMC6718414; doi:10.1038/s41467-019-11945-9)
Supplement: Supplementary file 1 — Supplementary Information [file 41467_2019_11945_MOESM1_ESM.pdf]

Supplementary Table 1. Cluster analysis of lipid metabolism proteins (from Fig. 4b)

	**AT-1$^{S113R/+}$**		**AT-1 sTg**	
Gene Symbol	proteome	acetyl-proteome site	proteome	acetyl-proteome site
Aacs	no	K640	no	no
Abat	yes	no	no	K452
Abca1	no	no	yes	no
Abcd4	no	no	yes	no
Abcd3	no	no	yes	no
Acaa1a	yes	K79; K198; K209	yes	K79
Acaa1b	no	K209	yes	no
Acaa2	yes	K234	no	no
Acaca	no	K1928	yes	no
Acacb	no	no	yes	no
Acadl	yes	no	yes	no
Acadm	no	K271	no	K271
Acads	no	K72	yes	no
Acadsb	yes	no	no	no
Acadvl	no	no	yes	no
Acat1	yes	no	yes	K400
Acat2	no	no	yes	K341
Acox1	no	K643; K500	yes	no
Acox2	yes	no	yes	no
Acsl1	yes	K649	yes	K655
Acsl3	no	no	yes	no
Acsl4	no	no	yes	no
Acsm1	yes	K527	no	K400; K527
Acsm5	yes	no	yes	no
Acss2	no	no	yes	no
Adh1	yes	K40; K85	yes	K85
Adh5	no	K101; K323	yes	no
Aldh2	no	K75	yes	no
Aldh3a2	no	no	yes	no
Aldh5a1	no	no	yes	no
Aldh6a1	no	no	yes	K76
Aldh7a1	no	no	no	K452; K514
Aldh9a1	no	K30	yes	K30
Amacr	no	no	yes	no
Apoa1	yes	K227	no	no
Apob	no	no	no	K196

Bckdha	yes	no	no	no
Bdh2	no	no	yes	no
Cat	no	K477	yes	no
Cpt1a	yes	no	no	no
Cpt2	no	no	yes	no
Crat	no	no	yes	K364
Cyp4a10	no	no	yes	no
Dld	no	K417	no	K417
Ech1	no	K230	no	no
Echdc1	no	K266	no	K266
Eci1	yes	K206	no	no
Eci2	no	no	yes	no
Ehhadh	no	no	yes	K673
Elovl1	no	no	yes	no
Fabp1	no	K6	no	no
Gcdh	no	K170; K377	no	K170
Got2	no	K94	yes	no
Hadh	no	no	no	K192
Hadha	no	K455	no	no
Hadhb	no	no	yes	no
Hibch	yes	no	no	no
Hmgcl	yes	no	no	no
Hmgcs1	no	no	yes	no
Hsd17b12	no	no	yes	no
Hsd17b4	no	K84	yes	K84; K301
Idh1	no	no	yes	no
Ldha	no	K224	yes	K228
Mttp	no	no	no	K609
Pcca	no	K294	yes	K146
Pccb	no	K491	yes	no
Pecr	no	no	yes	no
Pex1	no	K410	no	K410
Pex19	no	no	yes	no
Pex6	no	no	yes	no
Prdx1	no	no	yes	no
Prdx5	no	no	no	K70
Scp2	no	no	yes	K438
Slc25a20	no	K202	yes	no
Slc27a2	yes	no	yes	no
Suclg2	no	no	no	K355
Tecr	no	no	yes	no

Supplementary Table 2. Cluster analysis of mitochondria-related proteins (from Fig. 4c)

	AT-1$^{S113R/+}$		AT-1 sTg	
Gene Symbol	proteome	acetyl-proteome site	proteome	acetyl-proteome site
Acaa1a	yes	K79; K198; K209	yes	K79
Acaa1b	no	K209	yes	no
Acadl	yes	no	yes	no
Acadm	no	K271	no	K271
Acly	no	no	no	K968
Aco1	no	K400; K610; K587	no	K400; K587; K732
Acox1	no	K643; K500	yes	no
Acox2	yes	no	yes	no
Acsl1	yes	K649	yes	K655
Acsl3	no	no	yes	no
Acsl4	no	no	yes	no
Apoa1	yes	K227	no	no
Apoa2	yes	no	yes	no
Atp2a1	no	no	yes	no
Atp2a2	no	no	yes	K460
Atp5l	no	no	yes	no
Atp6v0a1	no	no	yes	no
Atp6v1a	no	K220	yes	no
Atp6v1e1	no	no	no	K104
Bid	no	no	yes	no
Casp7	no	no	yes	no
Casp8	no	no	yes	no
Cox4i1	yes	no	yes	no
Cox6b1	no	no	yes	K13
Cpt1a	yes	no	no	no
Cpt2	no	no	yes	no
Cs	no	K321	yes	no
Cyc1	no	no	yes	no
Cycs	no	no	yes	no
Cyp4a10	no	no	yes	no
Dlat	no	K363	no	K363
Dld	no	K417	no	K417
Ehhadh	no	no	yes	K673
Fabp1	no	K6	no	no
Fabp5	no	no	yes	no
Idh1	no	no	yes	no

Idh3a	no	K134	no	K134
Lhpp	no	K44	yes	no
Mapk1	no	no	yes	no
Mapk3	no	K33	no	K33
Mdh1	yes	no	yes	no
Me1	no	K26	yes	K204
Ndufa12	no	no	yes	no
Ndufa2	yes	no	no	no
Ndufa4	no	no	yes	no
Ndufa5	yes	no	no	no
Ndufa8	no	no	yes	no
Ndufa9	no	K118	no	no
Ndufb5	no	no	no	K176
Ndufb7	yes	no	no	no
Ndufb8	no	K176	no	K176
Ndufb9	no	no	yes	no
Ndufs1	no	no	yes	no
Ndufs3	no	no	yes	no
Ndufs4	no	no	yes	no
Ndufs8	no	K51	no	K51
Ndufv2	yes	no	no	no
Ogdh	no	K981	no	no
Pck1	no	no	yes	no
Pdha1	yes	no	no	no
Pdhb	no	no	yes	no
Ppa1	no	no	yes	no
Scp2	no	no	yes	K438
Sdha	yes	no	yes	no
Sdhb	yes	K235	no	no
Slc27a2	yes	no	yes	no
Slc27a5	no	no	yes	K500
Suclg2	no	no	no	K355
Uqcrb	no	K78; K88	yes	K45
Uqcrc1	no	no	yes	no
Uqcrfs1	yes	no	no	K251

Supplementary Table 3. Isotopic distribution of metabolites

Compound (isotopes quantified)	*m/z quantified (Th)*	*Retention time (min)*	*Formula of feature quantified*	*Method analyzed*
Citrate (M+0 – M+8)	375.1108	15.75	$C_{14}H_{27}O_6Si_3$	GC-MS
Alpha-ketoglutarate (M+0 – M+7)	304.1034	13.28	$C_{11}H_{22}NO_5Si_2$	GC-MS
Fumarate (M+0 – M+6)	245.0658	10.70	$C_9H_{17}O_4Si_2$	GC-MS
Malate (M+0 – M+6)	245.0660	12.31	$C_9H_{17}O_4Si_2$	GC-MS
Aspartate (M+0 – M+5)	232.1184	12.69	$C_9H_{22}O_2NSi_2$	GC-MS
Glutamate (M+0 – M+7)	363.1709	13.76	$C_{14}H_{33}O_4NSi_3$	GC-MS
Glutamine (M+0 – M+7)	347.1638	15.33	$C_{13}H_{31}O_3N_2Si_3$	GC-MS
Glucose (M+0 – M+8)	361.1681	17.06	$C_{15}H_{33}O_4Si_3$	GC-MS
