# Supplementary material for: Acetyl-CoA flux regulates the proteome and acetyl-proteome to maintain intracellular metabolic crosstalk
Source: Nat Commun. 2019 Sep 2;10:3929. doi: 10.1038/s41467-019-11945-9 (PMC6718414; doi:10.1038/s41467-019-11945-9)
Supplement: Supplementary file 4 — Reporting Summary [file 41467_2019_11945_MOESM4_ESM.pdf]

nature research

Corresponding author(s): Luigi Puglielli
Last updated by author(s): 7/23/2019

Reporting Summary

Nature Research wishes to improve the reproducibility of the work that we publish. This form provides structure for consistency and transparency in reporting. For further information on Nature Research policies, see Authors & Referees and the Editorial Policy Checklist.

Statistics

For all statistical analyses, confirm that the following items are present in the figure legend, table legend, main text, or Methods section.

n/a	Confirmed	
☐	☒	The exact sample size (*n*) for each experimental group/condition, given as a discrete number and unit of measurement
☐	☒	A statement on whether measurements were taken from distinct samples or whether the same sample was measured repeatedly
☐	☒	The statistical test(s) used AND whether they are one- or two-sided<br>*Only common tests should be described solely by name; describe more complex techniques in the Methods section.*
☒	☐	A description of all covariates tested
☐	☒	A description of any assumptions or corrections, such as tests of normality and adjustment for multiple comparisons
☐	☒	A full description of the statistical parameters including central tendency (e.g. means) or other basic estimates (e.g. regression coefficient) AND variation (e.g. standard deviation) or associated estimates of uncertainty (e.g. confidence intervals)
☐	☒	For null hypothesis testing, the test statistic (e.g. *F*, *t*, *r*) with confidence intervals, effect sizes, degrees of freedom and *P* value noted<br>*Give P values as exact values whenever suitable.*
☒	☐	For Bayesian analysis, information on the choice of priors and Markov chain Monte Carlo settings
☒	☐	For hierarchical and complex designs, identification of the appropriate level for tests and full reporting of outcomes
☒	☐	Estimates of effect sizes (e.g. Cohen's *d*, Pearson's *r*), indicating how they were calculated

Our web collection on statistics for biologists contains articles on many of the points above.

Software and code

Policy information about availability of computer code

Data collection	Data was collected using NIS-Elements Ar, Seahorse Wave software v 2.4, MATLAB, ThermoFisher TraceFinder, and Image Capture AMT v7.0.0.130
Data analysis	Statistical analyses were performed using Graphpad Prism v7.02, Microsoft Excel, and R v3.5.1. Other data analysis software included: Imaris imaging software (Bitplane, Oxford Instruments), Proteome Discoverer 2.1 engine, Byonic search engine, MaxQuant v1.6.1 software, Spectronaut v10, and Perseus v1.5.8.5.

For manuscripts utilizing custom algorithms or software that are central to the research but not yet described in published literature, software must be made available to editors/reviewers. We strongly encourage code deposition in a community repository (e.g. GitHub). See the Nature Research guidelines for submitting code & software for further information.

Data

Policy information about availability of data

All manuscripts must include a data availability statement. This statement should provide the following information, where applicable:

- Accession codes, unique identifiers, or web links for publicly available datasets
- A list of figures that have associated raw data
- A description of any restrictions on data availability

The mass spectrometry proteomics data that support the findings of this study have been deposited in the ProteomeXchange Consortium via the PRIDE partner repository with the accession code PXD013736. The acetyl-proteomics data that support the findings of this study have been deposited to the ProteomeXchange Consortium via the MassIVE partner repository with the accession code PXD014013. The metabolomics data that support the findings of this study have been deposited in the MassIVE repository with the accession code MSV000083885. The R script that was used to process the acetyl-proteomics data have been deposited on Github with the identifier (http://doi.org/10.5281/zenodo.3238525). The authors declare that all other data supporting the findings of this study are available within the paper and its Supplementary Data/Tables.

Field-specific reporting

Please select the one below that is the best fit for your research. If you are not sure, read the appropriate sections before making your selection.

☒ Life sciences ☐ Behavioural & social sciences ☐ Ecological, evolutionary & environmental sciences

For a reference copy of the document with all sections, see nature.com/documents/nr-reporting-summary-flat.pdf

Life sciences study design

All studies must disclose on these points even when the disclosure is negative.

Sample size	No sample size calculation was performed. Based on our previous publications, n=4 was used for proteomic and acetylomic analyses. By convention, for all other experiments n=4-8 and the specific sample size was reported throughout.
Data exclusions	No data was excluded.
Replication	With the exception of the proteomics and acetyl-proteomics, results were confirmed from experiments conducted at least 2 independent times.
Randomization	Mice were randomly placed into groups for diet studies (HCD vs. control). Animals from multiple litters were used to exclude variation among litters, and wild type littermates were used as controls in all experiments. Otherwise, randomization did not occur because genotype knowledge was necessary to perform experiments.
Blinding	Experiments were not blinded since animal phenotypes were easily distinguishable. For lipid serum analysis, investigators were blind to the genotype.

Reporting for specific materials, systems and methods

We require information from authors about some types of materials, experimental systems and methods used in many studies. Here, indicate whether each material, system or method listed is relevant to your study. If you are not sure if a list item applies to your research, read the appropriate section before selecting a response.

Materials & experimental systems

n/a	Involved in the study	
☒	☐	Antibodies
☒	☐	Eukaryotic cell lines
☒	☐	Palaeontology
☐	☒	Animals and other organisms
☒	☐	Human research participants
☒	☐	Clinical data

Methods

n/a	Involved in the study	
☒	☐	ChIP-seq
☒	☐	Flow cytometry
☒	☐	MRI-based neuroimaging

Animals and other organisms

Policy information about studies involving animals; ARRIVE guidelines recommended for reporting animal research

Laboratory animals	This study involved: mouse; C57BL/6; male; 6 months (AT-1 S113R/+) and 3 months (AT-1 sTg).
Wild animals	The study did not involve wild animals.
Field-collected samples	The study did not involve samples collected from the field.
Ethics oversight	All animal experiments were carried out in accordance with the NIH Guide for the Care and Use of Laboratory Animals and were approved by the Institutional Animal Care and Use Committee of the University of Wisconsin-Madison.

Note that full information on the approval of the study protocol must also be provided in the manuscript.
